# An Updated Overview of Almond Allergens

**DOI:** 10.3390/nu13082578

**Published:** 2021-07-27

**Authors:** Mário Bezerra, Miguel Ribeiro, Gilberto Igrejas

**Affiliations:** 1 Department of Genetics and Biotechnology, University of Trás-os-Montes and Alto Douro, 5000-801 Vila Real, Portugal; mariojbezerra02@gmail.com (M.B.); jmribeiro@utad.pt (M.R.); 2 Functional Genomics and Proteomics Unity, University of Trás-os-Montes and Alto Douro, 5000-801 Vila Real, Portugal; 3 LAQV-REQUIMTE, Faculty of Science and Technology, University Nova of Lisbon, Caparica, 2829-516 Lisbon, Portugal

**Keywords:** food allergy, almond, almond allergens, nutrition

## Abstract

Tree nuts are considered an important food in healthy diets. However, for part of the world’s population, they are one of the most common sources of food allergens causing acute allergic reactions that can become life-threatening. They are part of the Big Eight food groups which are responsible for more than 90% of food allergy cases in the United States, and within this group, almond allergies are persistent and normally severe and life-threatening. Almond is generally consumed raw, toasted or as an integral part of other foods. Its dietary consumption is generally associated with a reduced risk of cardiovascular diseases. Several almond proteins have been recognized as allergens. Six of them, namely Pru du 3, Pru du 4, Pru du 5, Pru du 6, Pru du 8 and Pru du 10, have been included in the WHO-IUIS list of allergens. Nevertheless, further studies are needed in relation to the accurate characterization of the already known almond allergens or putative ones and in relation to the IgE-binding properties of these allergens to avoid misidentifications. In this context, this work aims to critically review the almond allergy problematic and, specifically, to perform an extensive overview regarding known and novel putative almond allergens.

## 1. Introduction

Food allergies are a concerning issue affecting the worldwide population, and their prevalence has been increasing for the last couple of decades [1,2,3]. For example, in the United States, around twenty-six million adults [4] and six million children [1] suffer from this condition. Although there is no cure to food allergies and food avoidance is considered the best strategy, vast research has been made in this area and potential therapies can be generally divided into two categories: allergen non-specific such as the use of monoclonal antibodies and allergen specific where the treatment is performed using recombined or native food antigens [5]. However, less commonly, adverse side effects can range from mild to anaphylaxis or eosinophilic esophagitis [6] and due to their unpredictable character [7], new and innovating therapies must be pursued.

For scientific research to go further, food allergy, allergic diseases and allergens must be firstly identified and characterized. For allergens, when new ones from specific species are identified, a distinctive name is given by the WHO/IUIS Allergen Nomenclature Sub-Committee alongside the additional information about it. A vast number of allergens from more than one hundred and sixty species have been identified and most of them belong to a restricted number of protein families. Among these, the (1) tryp_alpha_amyl protein family includes the higher number of known food allergens, which includes, for example, lipid transfer proteins (LTPs) and 2S albumin seed storage proteins; (2) cupin_1 protein family including the 7S vicilin seed storage proteins and the 11S legumin, and the (3) profilin family comprising profilins, are the most prominent ones [8]. In almonds, several proteins of these protein families have been already identified as allergens, namely Pru du 6 (11S globulin legumin-like protein), Pru du 4 (profilin) and Pru du 3 (nonspecific LTP) and several other proteins belonging to other protein families and/or that do not have a name attributed by the Allergen Nomenclature Sub-Committee.

Great attention has already been devoted to this topic [8,9,10] and here we intend to present a comprehensive and updated overview of almond allergens, namely the description of Pru du 10, the most recent almond allergen to be added to the WHO-IUIS list of allergens. We also reviewed the legal framework of the European Union and the United States concerning food allergies and labelling, and the methods currently available for the detection and quantification of almond allergens in food products. All these topics combined offer a wide, updated, and comprehensive narrative about almond allergies and allergens. With that, this review aims to provide easy access to updated information about almond allergies to researchers, clinicians, and patients to be applied in their respective manners.

### Methods

The research documents analyzed in this work were extracted from the PubMed and Elsevier Scopus online databases collecting academic documents, both including keywords such as ‘almond’, ‘almond allergy’ or ‘almond allergens’ or other topics considered relevant. Only publications in English were included. The articles from the search were assessed according to document type, language, and inclusion in subject category. They were further analyzed, and the results were used to write this review.

## 2. Food Allergy

By definition, a food allergy is “an adverse food health effect arising from a specific immune response that occurs reproducibly on exposure to a given food” [11]. It is also important to clarify that the immune reaction is key, otherwise food allergies could probably be described as food intolerances, which are a non-immune response but may reproduce food allergy clinical symptoms [12].

Evidence that shows global variation of food allergies as well as changes in their prevalence associated with migration [13] are increasing the interest on the epidemiological strand of food allergies and may promote hypothesis for why food allergy is a rising issue in some parts of the world and not in others [14]. Some authors proposed various hypotheses on the increasing prevalence of food allergy in association with geographical sites; the most accepted ones were hygiene increases, which have led to less pathogen exposure, changes in the human microbiome, avoidance of certain allergens in the early stages of life causing allergen exposure reduction, obesity, diets lacking antioxidants and vitamin D deficiency [15,16].

Tree nuts are one of the Big Eight food groups among peanut, milk, shellfish, soy, wheat, egg and fish which are responsible for more than 90% of food allergy cases in the United States [8] and, in particular, the number of people sensitized to tree nuts and peanuts has been growing concerningly in Europe and the United States [17]. In this group of foods, almond and peanut allergies are persistent and normally severe and life-threatening in opposition to allergies caused by milk or eggs, which are normally mild and transient [18,19,20].

Tree nut allergy prevalence data is very limited and is even more limited for a specific nut species such as almonds [10]. However, it is known that tree nut allergy rates vary according to geographical regions, ethnic differences, and dietary habits [21]. 

### 2.1. Molecular Pathway of Immunoglobulin E-Mediated Food Reaction

Food allergies can arise through several immunological mechanisms that lead to a reaction to food allergens. The most common mechanism of food allergy expression is a hypersensitivity manifestation where specific Immunoglobulin E (IgE) antibodies interact with mast cells and basophils leading to a rapid physiological response [22]. Usually, food allergy symptoms appear nearly immediately, or a few minutes later after food ingestion, however in exceptional cases it could take several hours for the symptoms to manifest [23].

**Figure 1:**
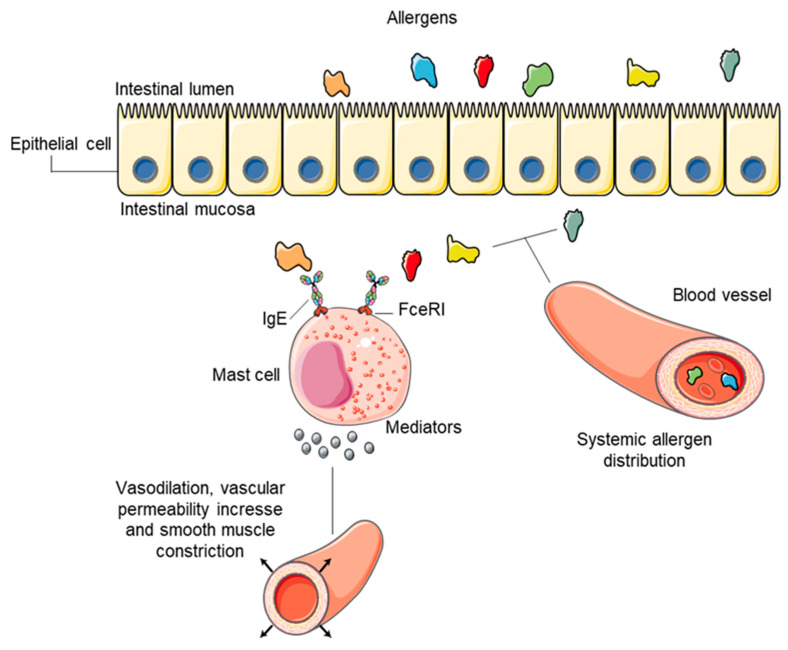
General mechanisms of IgE mediated response to food allergens. Interaction between food allergens and IgE and its high-affinity FC receptor (FCER1) on basophils in circulation or mast cells present in mucosal tissues leading to their activation and consequent physiological response. Adapted from Renz, Allen, Sicherer, Sampson, Lack, Beyer and Oettgen [22]. Adapted with permission from Ref. [22]. Copytright 2018. Springer Nature.

In people with food allergy disorders, the absorption process of allergens in the intestinal epithelium and consequent access to the bloodstream and mucosa is increased [24]. When food allergens are ingested, an interaction occurs between them and IgE and its high-affinity fragment crystallizable receptor (FCER1) on basophils in circulation, or mast cells present in mucosal tissues leading to their activation (Figure 1). FCER1 crosslinking leads to a signaling cascade where tyrosine protein kinase SYK will promote exocytosis of granules containing mediators of hypersensitivity such as histamine, chymase and tryptase [22]. This process together with the synthesis of lipid metabolites such as prostaglandins, leukotrienes and platelet-activating factor (PAF) [25] will result in physiological responses such as the activation of nociceptive nerves that promote itching and soft muscle constriction, vasodilation, higher vascular permeability and, in the most severe cases, anaphylaxis [26].

Although this is the generic mechanism after food ingestion, non-IgE mediated reactions such as the inflammatory process subjacent to eosinophilic esophagitis [27] can also occur [27,28]. The physiological response is dependent of the kind of mediators released by the mast cells and basophils but is also dependent on tissue location where these mediators would act. These two factors combined will directly influence the physiological response. [22].

### 2.2. Legal Framework

There are several regulatory frameworks for food allergen labeling according to countries or regions that differ significantly around the world due to the priority level that each jurisdiction applies to specific allergens. The criteria for the development of the allergen’s priority list and the standards for the addition or removal of allergens from the regulations differ and they are often unclear [29].

The Regulation (EU) No. 116/2011 sets the regulation on food labelling, forbidding misleading consumers and any claims that a certain food, such as almonds, can prevent, treat, or cure human diseases cannot be made. Moreover, nutritional and allergen information must be highlighted in the list of ingredients and included in non-packed foods or any product where they are used as ingredient, with the punishment of being withdrawn from the market.

Regulation (EU) No 1169/2011 of the European Parliament and of the Council of 25 October 2011 on the provision of food information to consumers states that allergens should be indicated in the list of ingredients with a clear reference to the name of the substance or product causing allergies or intolerances and should be emphasized through a typeset that clearly distinguishes it from the rest of the list of ingredients, for example by means of the font, style, or background color. In this list of substance or product causing allergies or intolerances nuts are included, with a clear reference to almonds, hazelnuts, walnuts and others, cereals containing gluten, crustaceans, eggs, fish, peanuts, soybeans, milk, celery, mustard, sesame, lupin, mollusks, and products from each one.

In the United States, food labelling requirements are quite similar to the ones applied in the European Union, where the Food Allergen Labeling and Consumer Protection Act of 2004 states that any food source containing a major food allergen, or protein derived from them, should be printed right next to the ingredient list, and specifically have the word “contains” before it. The term “major food allergen” refers to milk, egg fish, crustacean shellfish, tree nuts (like almonds, pecans, or walnuts), wheat, peanuts, and soybeans, however any highly refined oil derived from any of the previous foods and products derived from those oils are considered exceptions. 

For the appliance of the food labelling requirements, it is important to defined threshold values which correspond to the minimal concentration of a specific food allergen in a food able to trigger any reaction in a sensitized individual. However, is very difficult to establish a threshold, since they vary according to the individual/population, the allergens itself and the consequent food processing [30]. To get there, wide population tests and data are needed. For almonds, currently no thresholds are established [10], which shows a clear sign that further investigations and regulations are imperative.

## 3. Almond

One of the most important foods in human nutrition are tree nuts, namely due to their excellence in terms of taste as well as their versatility to be used combined with other foods and, more recently, their potential health benefits. All these characteristics mean that tree nuts are consumed all around the world in the most various of forms, according to the availability in the region and the populational habits [9,31].

The almond (*Prunus dulcis* Mill.) is a member of the *Rosaceae* family and is considered a native plant from Minor Asia [32], being one of the oldest nut trees cultivated worldwide with special relevance in the Mediterranean warm-arid countries [33,34], namely the Apulia region on southern Italy [35]. Among tree nuts, almonds present as one of the most important nuts, which is very noticeable in tree nut production data around the world (Walnut 3663, Almond 3183 and Hazelnut 864 ktons/year; [36]). Furthermore, its nutritional properties should be highlighted; high levels of mono and polyunsaturated fatty acids, phytosterols and a low glycemic index are associated with reduction of some risk factors for cardiovascular disease and diabetes [37,38,39,40]. It has also been described as having antioxidant and inflammatory activities due to its polyphenol content, including flavonoids, hepato and neuroprotective potential and, perhaps the most known, cholesterol-lowering properties [41,42,43,44]. Also, almond derived products such as their oils have demonstrated both antibacterial and antifungal capabilities [45] which makes almond a product of great interest both to the consumer and producer. 

Regarding almond cultivars, European commercial cultivars such as the Spanish Marcona, Glorieta, Masbovera, Guara and Francolí cvs. and the French Ferrastar, Ferraduel and Ferragnès cvs. are the main ones produced in Europe. In the United States, the most widely produced almond variety in Nonpareil cv. represents near half of the production. On other hand, in Portugal there is a mix of traditional and local varieties such as Amendoão, Pegarinhos, Casanova and Refego cvs. [46,47]. However, in a study testing three almond varieties Nonpareil, Mission and Carmel against eight almond allergic patient’s sera, no significant differences were found. New similar research must be conducted to correctly evaluate the allergic potential of each variety of interest [48].

Along with the almond nutritional value comes the agronomical properties of different cultivars. For example, Bolling et al. [49] described that the individual polyphenols synthesis was only due to the cultivar itself, however total polyphenols and antioxidant activity were significantly dependent on both genotype and environmental growing conditions. Pursuing this point of view, Summo et al. [50] performed a study aiming to determine if either the cultivar or harvest time influence the chemical composition of the fruit. From this, the team concluded that, in fact, harvest time and genotype both have a strong influence on the fruit nutritional value.

### 3.1. Almond Allergy

Nut allergy is associated with clinical symptoms that can range in severity from mild to life-threatening, and in this sense when a patient is diagnosed with an allergy to a certain nut it is often advised to avoid the consumption of the entire group [51,52]. 

Epidemiologically speaking, almond allergies have the fourth highest prevalence among the tree nuts allergies [53]. Looking at the specific cases of the United States, Korea, United Kingdom, Mexico and Sweden, almonds present the third most common tree nut to cause allergies in the United States [10], and between 9% and 15% of people pre-sensitized to tree nuts also report allergy to almonds [54]. In a study performed in a group of 134 Korean patients with previous reports of food allergies, 11.2% also reported almond allergies. Among them, 16.3% were between 19 to 29 years old, 13% in the 40–49 age group and 9.1% in the 50–59 group. Also, the same study reported that sensitivity to almonds is lower in females, with 9.8% compared to males at 13.5% [55]. In the United Kingdom, in pre-sensitized individuals, almonds represent the most common tree nut allergy, with 22% to 33% of the cases [54,56]. The higher rate of sensitization to almonds was reported in a study performed in Mexico City, reporting a 43% rate in older children with ages comprised between 6 and 17 years old [57]. A cross-sectional enquiry made in Sweden with 1042 responses from individuals between 17 and 78 years old concluded that near 32.5% of adults had food hypersensitivity and 3% were sensitive to almonds [58].

Almond allergy can cause several clinical responses. The Oral Allergy Syndrome (OAS) is a pollen-food syndrome that produces mild oral symptoms in cases of pollen sensitization triggered by nuts. Although it hardly causes anaphylaxis, it can happen in the direct confrontation of serum sIgE with PR-10 homologous [59]. Another common clinical response is allergic rhinitis, that has been associated with almond allergies in a study performed in southern Taiwan with a group of 216 individuals with ages comprised between 2 and 93 years old. Most of these people had respiratory and cutaneous symptoms, and the study reported a 36.97% prevalence of allergic rhinitis caused by almonds in the group of the non-sensitized patients. Besides allergic rhinitis, asthma has been associated with almonds with a prevalence of 7.4% in the non-sensitized nut group and 13.70% in the sensitized one. Also in Taiwan, it was reported that almonds were responsible for 42.47% of atopic dermatitis cases in a group of 33 nut sensitized individuals [60]. Other symptoms can emerge, such as gastrointestinal ones. In a group of 1024 sensitive individuals, 15% reported these, and from those, 2.7% were due to almonds [58].

Regarding strategies for prevention and therapy for an almond allergy, the main method is dietary avoidance. Individuals sensitive to almonds should take special attention looking at packages and labels to prevent the ingestion of almond or almond-based products [59]. However, there are some strategies that seem to prevent the development of almond allergies, namely the premature consumption of almonds during infancy or even during pregnancy, or lactation also showed a positive impact on its prevention [61]. Moreover, there is evidence that about 10% of tree nut allergies are outgrown by young individuals who develop tolerance due to the rise of T regulatory cells and the consequent reduction of allergen specific IgE [62]. Immunotherapy, a food allergen-specific therapy, which refers to the administration of gradual and increasing doses of an antigen over a certain time [63,64], is considered as a solid option since in the majority of cases the side effects are mild, such as itching and, if successful, immunotherapy can induce desensitization and less commonly sustained unresponsiveness, also known as tolerance [5]. Moore, Stewart and Deshazo [5] believe that tolerance induced by immunotherapy with or without the administration of monoclonal antibodies could significantly shift the allergic diseases field.

Cross reactivity between almonds and other sources of allergens is a well-known problem and there are some of these associations (summarily described in Table 1) already described. 

**Figure 2:**
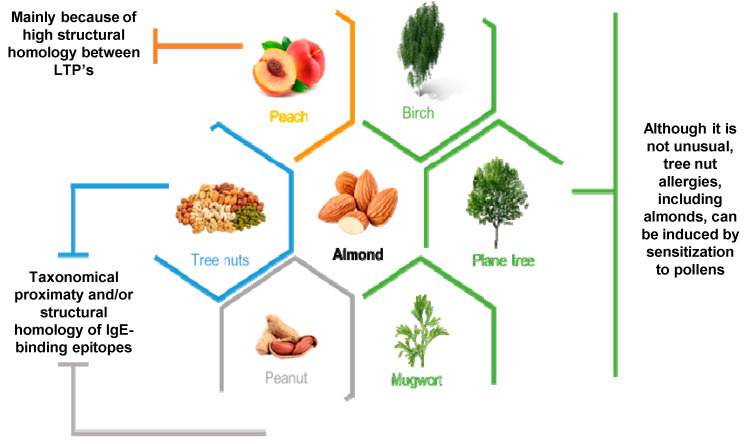
Most associated allergic cross-reactions with almonds. In orange, allergic cross reactions between almonds and peaches are most commonly due to high structural homology between allergic LTP’s present in the Rosaceae family that both belong to; in blue and grey, it is still unclear if cross reactivity between almonds and other tree nuts groups and peanuts is a consequence of taxonomical proximity and/or high structural homology of IgE-binding epitopes; finally, in green are represented three different pollens which, although it is not usual, when sensitized to them allergies to tree nuts such as almonds could be induced.

Nevertheless, it is still unclear if the taxonomic proximity between tree nuts groups and peanuts is a key factor for the cross-reactivity between these two, or it comes from the high structural homology of IgE-binding epitopes [70,71]. In general, tree nut allergies are caused by non-pollen-mediated food sensitization, however, in cases such as with almonds and hazelnuts, sensitization to plane tree pollen, birch pollen or mugwort pollen may induce allergies [72,73] such as those schematically represented in Figure 2. On the other hand, tree nut allergy cross reaction is highly related to botanical family associations which, for almonds, is common regarding cross-reactivity between other members of the *Rosaceae* family [74,75]. Furthermore, within the *Rosaceae* family, a strong source of cross-reaction lies in the structural homology between allergic lipid-transfer proteins (LTP’s). Specifically, in the tree nut group, almond Pru du 3, chestnut Cas s 8, hazelnut Cor a 8 and walnut Jug r 3 are the most predisposed to show cross-reactivity. Besides these, peach Pru p 3 holds higher IgE-binding affinity and a higher number of epitopes compared to other LTP’s, which results in the fact that a peach is a primary sensitizer to LTP’s [65] and makes it a strong cause for cross-reactivity to other plants, including nuts like almonds [76]. Other studies performed by Kewalramani et al. [77] showed extensive IgE cross-reactivity between almonds and apricot seeds, and that there may exist some cross-reactive proteins with pine nut, pecan, walnut, and sunflower seeds.

### 3.2. Almond Allergens

To date, ten groups of almond allergens have been identified, namely: Pru du 1, Pru du 2, Pru du 2S albumin, Pru du 3, Pru du 4, Pru du 5, Pru du 6 (amandin), Pru du γ-conglutin, Pru du 8 and Pru du 10. From these groups, only Pru du 1, Pru du 2, Pru du 2S albumin and Pru du γ-conglutin are not included in the WHO-IUIS list of allergens. Their corresponding biochemical names, biological functions, GenBank nucleotides and UniProt annotations, molecular weight, food processing effects and clinical relevance are summarized in Table 2.

#### 3.2.1. WHO/IUIS Designated Almond Allergens

##### Pru du 6 (Amandin)

Pru du 6 or amandin is the most well and widely studied almond allergen according to its biochemical function and molecular structure [78,79,80,81]. It was first reported as an allergen in 1999 [77] but was only recognized in 2010 and added to the WHO-IUIS database.

Biochemically, amandin, also known as almond major protein (AMP), is a member of the cupin superfamily, namely the 11S seed storage globulin family [51,52]. Globulins are very abundant proteins in legumes and tree nuts, and in almonds they correspond to roughly 65% of total almond protein content [9]. 

As an allergen, Pru du 6 have been associated with severe allergic reactions [80]. Studies on the Pru du 6 isoforms, Pru du 6.01 and Pru du 6.02, showed that the 6.01 isoform is more broadly recognized than the 6.02 isoform. In addition, its denaturation had only slightly effects on IgE-binding intensity in sensitive subjects [82]. In fact, Pru du 6 polypeptides are highly resistant to heat treatment, which is one of the most common strategies to decrease or even eliminate the allergenic potential of foods. Due to its heat resistance, contamination of food with Pru du 6 polypeptides presents a serious threat to sensitized patients [83]. On the other hand, some experiments using in vitro models of gastrointestinal digestion suggested that this allergen is sensitive to pepsin but, interestingly, when almond flour is added to other foods, pepsin’s action on Pru du 6 is a lot less effective [84]. Holden et al. [85] suggested that the reaction between Pru du 6 and α-conglutin from lupine, another 11S globulin, may be the cause of it.

##### Pru du 5 (60S Acidic Ribossomal Protein P2)

Pru du 5, also known as 60S acidic ribosomal protein P2, is encoded by *P. dulcis* 60S acidic ribosomal protein gene and was included in the WHO/IUIS allergen list in 2007. This name comes from the fact that this allergen is an 11 kDa protein which is a member of the 60S large subunit of the eukaryotic 80S ribosomes [8], and its biological function is related to protein biosynthesis. Pru du 5 is considered a major almond allergen due to the presence of specific IgE antibodies in 50% of sensitized patients’ sera [86].

This allergen can exist as a complex with other ribosomal components/proteins or in its free state [65], with the ability to form homodimers and oligomers [72,74]. On the allergenicity front, this data is very important because oligomerization gives the allergen the capability of cross-linking IgE antibodies on mast cells and/or basophils surfaces, even if the recognition is made from a single epitope of the allergen [8].

Although being considered a major allergen and present in the WHO-IUIS allergen list, many authors believe that this classification must be supported by more studies concerning the IgE reactivity of allergic patients’ sera to this allergen [9,10]. Also, studies regarding the biochemical and immunological properties of Pru du 5 in its natural state as an allergen are lacking [8], leading to the conclusion that newer and tougher studies are needed. 

##### Pru du 3 (nsLTP)

Added to the WHO/IUIS database in 2009, Pru du 3 is a non-specific lipid transfer protein 1 (nsLTP1) belonging to the subfamily of nonspecific lipid transfer proteins (nsLTPs) [75]. This family includes proteins constituted by a hydrophobic core to ease lipid transference such as phospholipids, steroids, fatty acids, and glycolipids between membranes. Besides that, nsLTPs are also known as pathogenesis-related 14 (PR-14) proteins, a member of the prolamin superfamily [9,65], which actively participate in plant-defense mechanisms against fungal and bacterial pathogens and other environmental stresses [76].

In almonds we identified and characterized three nsLTPs [87] with identical molecular weights (9 kDa) and similar amino acid lengths: 117, 123 and 116 amino acids for Pru du 3.01, 3.02 and 3.03, respectively. In the three isoallergens, there are eight cysteine conserved residues, which allow the formation of four disulfide bonds [9].

Due to the typical accumulation of this protein family in outer epidermal layers, the peels are associated with stronger allergenicity compared with the pulps of the fruits in the *Rosaceae* family. Regarding allergenicity, this protein family is quite concerning because of its resistance to abrupt pH changes, pepsin digestion, thermal treatments, and the ability of restore folding structures and the consequent proprieties after cooling [88]. Cross-reactivity is also a major concern once the nsLTP family is characterized by a high level of conserved sequences and tridimensional structures allowing IgE recognition, which in turn results in cross-reactivity between species [76]. Furthermore, the Rosaceae fruits and seeds normally present nsLTP proteins, and with that comes a high probability of cross-reactivity between, for example, apples, peaches, cherries, apricots and almonds [89]. This latest evidence is the main reason why nsLTPs are included in the panallergens group—allergens ubiquitously spread throughout nature, showing a high level of conservation besides being from different and unrelated organisms [8]. 

##### Pru du 4 (Profilins)

Pru du 4 proteins are included in the profilin family and are encoded by the putative genes *Pru du 4.01* and *Pru du 4.02* [68] which, although present in different size fragments (1041 and 754 bp, respectively) encode two proteins with similar sequences (131 aa), molecular weights (roughly 14 kDa) and acidic properties (*p*I near 4.6) [9]. 

These proteins can establish high-affinity complexes with monomeric actin, leading to its polymerization into filaments. Once they are associated with actin, it is not surprising that profilin allergens are included in the panallergens group with Pru p 4.01 and Pru av 4 from peaches and sweet cherries, respectively, being the most similar and identical proteins (99 and 98%, respectively) in relation to almond profilins. In general, profilins seem to present moderate structural stability, and harsh conditions contribute to their denaturation and consequent loss of conformational structure. In almonds, Pru du 4 profilins are very difficult to detect by immunoblot screens because of their low levels and their labile character. Because almond profilins antibodies are detected in 44% of patients’ sera, they are classified as minor allergens [68].

##### Pru du 8

Pru du 8 is one of the latest allergens included in the WHO-IUIS database. This allergen was reactive in six of eighteen sera of almond allergic patients [10,84]. Biochemically speaking, Pru du 8 is characterized by a signature repeat of a CX_3_CX_10-12_CX_3_C (X being any amino acid), motif which is also related to the N-terminal or the signal peptide of some vicilins [90], and it was also reported to maintain antimicrobial function of some peptides derived from macadamia vicilin [91].

The first nomenclature attempt for this allergen was based on the sequencing of two short peptides of this allergen to reveal the identity of an IgE-reacting protein several years ago. Nevertheless, the result was a misidentification of this allergen as an almond 2S albumin because of the sequence alignment of the two peptide sequences and those in other 2S albumin proteins [92]. More recently, in silico investigations and bioinformatic analyses reopened the debate, naming this allergen as Pru du vicilin (almond 7S vicilin), although some authors believe in a second misidentification [8,93]. In fact, the authors claim that this misidentification is due to the similarity between the signal peptides of vicilins of other species and Pru du 8. Besides that, it is argued that some Pru du 8 orthologs present in the NCBI database, most of them predicted by automatic genome annotations, are incorrectly named as vicilin-like proteins due to the absence of the cupin signature domains of 7S vicilins [8,90].

All this controversy shows that further studies are needed to better elucidate the actual protein family of Pru du 8.

##### Pru du 10

To date, this allergen was the last one to be added to the WHO-IUIS database. This allergen corresponds to mandelonitrile lyase 2 (formerly hydroxynitrile lyase 2), which is a highly effective catalytic enzyme [87]. This allergenicity was recognized after allergic response to almond ingestion where thirteen of eighteen almond allergic patients were sensitized. Also, the Pru du 10.0101 isoallergen was identified and added to the WHO-IUIS allergen information.

Besides being identified in raw almond samples, this protein was also identified in digested samples, which may indicate that this allergen is able to overcome the digestion process [89]. Still, there is a lack of information regarding this allergen which clearly shows that more studies should address this issue. 

#### 3.2.2. Allergens Not Included in the WHO/IUIS Allergen List

There are two main processes to classify a protein as a food allergen, based on immunological data such as the IgE reactivity or based on sequence similarity with proteins of other species already considered allergens. For an allergen to be included in the WHO-IUIS database, immunological data is required and because of that, some authors defend that those which cannot be supported by it should hardly be assumed as an allergen. However, bioinformatic-based investigation is very important to promote further investigation and make aware the scientific and industrial community to the dangers of food allergens.

##### Pru du γ-Conglutin

The IgE and serological reactivity to Pru du γ-conglutins were not associated with any clinical symptoms and because of that, they are not recognized into standard clinical nomenclature [10].

After the report and characterization of conglutins in other fruits and seeds such as lupine [94], peanut [95], soybean [96] or cashew [97], in almonds an N-terminal peptide sequence of 25 aa belonging to a IgE binding protein with a molecular weight of 45 kDa was also identified, presenting around a 40% identity rate between the mature forms of γ-conglutin from wide and narrow-leafed lupine [92]. Moreover, with a high similarity, approximately 50%, between this almond protein and 7S globulin from soybean, this allergen was considered a vicilin (7S globulins) of the cupin superfamily [8,9]. Nevertheless, some authors do not agree with this classification, stating that γ-conglutin is not a vicilin due to its biochemical properties [8]. In particular γ-conglutin presents sequence and structural similarities with xyloglucan-specific endo-beta 1,4-glucanase inhibitors, however such glucanase inhibition properties are not related to the natural γ-conglutin due to is peptidase cleavage susceptibility [98].

The same authors believe that more studies regarding immunological and biochemical properties of this protein are needed, and the confirmation of this assumption would make this protein the first food allergen from this supposed protein family.

##### Pru du 1-PR-10 Protein (Pathogenesis Related-10 Protein)

Pathogenesis related proteins are a common group of proteins, generally upregulated in plants to promote defense mechanisms against pathogens such as viruses, bacteria or fungi and environmental factors [8]. The PR-10 family is related to the intracellular defense processes and the response to fungal and bacterial infections. Due to its function, there are numerous isoforms which promote different IgE-binding capabilities [89]. Furthermore, PR-10 proteins are constitutively expressed in different plant parts and usually are not related to other PR proteins [99]. They are commonly seen as pollen or food allergens [100,101] and because of that they can be considered as panallergens, being responsible for cross-reaction events [76].

Although there is no immunological data to support their classification as an allergen and the high similarity and identity between almond PR-10 proteins and the peach counterparts, which are known allergens (Pru p 1), almond PR-10 proteins are assumed as an allergen and named as Pru du 1 [76].

##### Pru du 2 (PR-5/Thaumatin-Like Protein)

This allergen group is also known as PR-5 or thaumatin-like proteins (TLPs) and are responsible for the biological response to pathogen infection, fungal proteins, and osmotic stress. The TLP’s group is known to be very resistant to proteases, heat-induced denaturation, and pH variations, possibly because of sixteen conserved cysteine residues which form eight disulfide bonds [89]. Several isoallergen genes have been identified which code for TLP, ranging in molecular weight from 23 to 27 kDa. Also, the isoallergens aminoacidic sequence length ranges from 246 aa to 330 [102].

Like PR-10 proteins, no immunological characterization of PR-5 almond proteins exists. Although, it is believed that these proteins are almond allergens due to the high sequence identity with Pru p 2, a peach allergen [103]. Moreover, due to their biochemical properties, traditional food-processing practices do not significantly influence these protein’s structure and characteristics, so they could affect sensitive patients [9]. 

##### Pru du 2S Albumin

Included in the prolamin superfamily, 2S albumins are an important group of seed storage proteins involved in seed growth and in defense related mechanisms [104,105]. Besides 2S albumin, the prolamin superfamily also includes other protein groups such as the nonspecific lipid transfer proteins (nsLTPs), prolamin storage proteins and α-amylase/trypsin inhibitors, which may indicate several cross-reactions [106].

2S albumins are thought to be somehow resistant to acidic pH enzyme digestion, particularly the albumins with proteolytic activity and surfactant denaturation effects. These conclusions come from the fact that is believed to this proteins cause sensitization along the intestinal tract, which could only be possible if the previous resistances were actually accurate [107].

As an allergen, the strongest data that lead to the classification of almond 2S albumins as almond allergens is the two short partial peptide sequences with high similarity with 2S albumins of other species [108] that, as discussed in Section 3.2.1, some authors believe to be a misidentification and really correspond to Pru du 8 proteins [8]. In fact, 2S albumins of other species, such as Ara h 2 (peanut 2S albumins) for example, are very potent allergens [109,110,111] and for this reason the assessment of whether these almond proteins are allergens or not is required and imperative.

### 3.3. Methods for Almond Allergens Detection

Most of the methods used for the detection of almond allergens are based in immunochemical properties, DNA techniques and, lately, in Mass Spectrometry (MS) approaches [9].

The immunochemical methods are based on the interaction between immunoglobulins and epitopes present in the target protein. For almond allergen detection, lateral flow devices (LFD), immunoblotting and especially Enzyme-Linked Immunosorbent Assay (ELISA), are very standard methods and the usual techniques for quantitative and qualitative detection of food allergens [114,115]. This comes from the fact that ELISA tests, for example, have enough sensitiveness for protein detection (in the orders of ppm), being the main advantage of the fast assessment, which is important for clinical purposes [115]. Several immunological commercial kits, such as the ones exemplified in Table 3, have been developed with the objective of delivering the most sensitive result in the shortest amount of time. As seen in the kit’s characteristics, ELISA-based methods provide more sensitive results, as their limit of detection is lower than the LFD-based kits. However, the assay time is longer for the ELISA cases. Taking this into consideration, the assay type should be taken into serious consideration, according to the situation that are supposed to be used.

Another possible approach, instead of looking directly for the protein itself, is the DNA-based method where an amplification is performed of the gene fragment responsible for encoding the allergen by Polymerase Chain Reaction (PCR), allowing quantitative and qualitative measurement using real-time PCR or endpoint PCR assays, respectively [10]. One of the advantages of these methods is that they rely on the detection of low quantities of almond DNA even after food processing, which could promote the degradation of some allergen proteins and therefore not be detected by immunological approaches [116]. However, the presence of the gene encoding the allergens does not imply its expression and, because of that, the synergistically use of DNA-based techniques and ELISA could overcome some of the drawbacks of both techniques [117].

Proteomics play a very important role in the food allergy problematic, firstly on a fundamental investigation basis to characterize allergens and further to their application in the diagnostic routines. Namely, a variety of tests and methods must be applied to characterize allergens according to their allergenic activities, purity and folding properties. Following this line of thought, SDS-Polyacrylamide Gel Electrophoresis (SDS-PAGE) is a reliable technique to determine purity, and following 2 Dimension (2D) electrophoresis, capillary electrophoresis or High-Performance Liquid Chromatography (HPLC) are great techniques to access individual isoforms and obtain more additional information in general. Further, MS techniques are powerful tools to determine protein molecular masses, being Matrix Assisted Laser Desorption Ionization (MALDI) and ElectroSpray Ionization (ESI), as the most commonly used [118,119]. MS techniques have been the most recent methods to be explored for qualitative and quantitative purposes [120,121]. For example, the isolation and characterization of Pru du 3 allergen was conducted using MS techniques where the full sequence was obtained by Liquid Chromatography ElectroSpray Ionization Orbitrap Mass Spectrometry (LC-ESI-Orbitrap-MS) [122]. Mass spectrometry has the advantage of ELISA tests which can directly identify proteins with a high sensitivity, and therefore could provide a direct risk evaluation and, besides that, can be used for the detection of multiple allergens simultaneously [122]. MS could be the chosen technique for a standard test; however, it is a relatively recent approach which demands expensive equipment and specialized personnel. At this standpoint, further improvements are required to allow easier access and profitable use by clinical facilities [10]. 

Another methodology under development is based on microarrays. Namely, allergen microarrays such as the MeDALL allergen-chip have been explored for the diagnosis and monitoring of allergies. The main advantages rely on the simultaneous detection of several allergens with a minimal amount of sera in a reduced time. The development of this chip has the purpose of monitoring IgE and IgG reactivity profiles against 170 allergens in sera collected from European birth cohorts. With that information, it would be possible to make a geographical association of clinical important allergens in different populations and track the progress of food allergy itself and would allow clinical therapies to act in a prophylactic and more personalized manner [123].

It is worth mentioning the basophil activation test (BAT) as a powerful method for tree nut allergy diagnosing [124]. This is an in vitro assay based on flowcytometry protocols that, essentially, allows the evaluation of activation and/or degradation levels of basophils upon the intentional contact with the pretended food allergens [125]. However, it also has some limitations, mainly because of the level of equipment required which makes difficult the use of this technique in small medical centers; this could be overcome with the use of specialized centers and with new research to lower the costs. On other hand, results have been shown that BAT assays have very strong performances and useful results, including multi-nut sensitizations and, because of that, medical infrastructures should take this test into consideration for these kinds of diagnostics [126].

## 4. Conclusions

Almond production has been increasing for the last years and is currently positioned as one of the most consumed tree nuts and one of the most likely to cause mild to severe allergic reactions. Worldwide data regarding the epidemiological standpoint of almond allergies is concerningly scarce. Without this kind of information, it is hard for governmental and medical institutions to establish personalized and efficient protocols and initiatives to mitigate this problem. 

On the other hand, a lot of almond proteins have been already described as potential allergens, although only a part of them have been recognized as allergenic and the authenticity of some designations have been questioned, mainly due to misidentification problems. It is expected that the development of suitable analytical methods for the efficient detection of food allergens and its characterization, for example supported by comprehensive proteomics approaches, will help in the validation of many of these proteins/allergens in the years to come.

For the near future, the develop of new techniques and the increasing usage of powerful ones like BAT should happen to take a step forward into the search for a more permanent solution. In the meantime, accurate characterization of ancient and local varieties should be made for the possible selection of hypoallergenic varieties, and breeding programs can be used for the development of varieties with hypoallergenic characteristics. Moreover, the effort of also evaluating almond-based products must be made to secure safety for the general consumer.

However, a long way is yet to be made and researchers, clinical institutions and governmental entities must work together to establish an efficient network covering all the aspects of almond allergies in order to better understand this problem and enable the development of new and more efficient preventive therapies.

## Figures and Tables

**Table 1:** Almonds’ most common cross reactions with other relevant sources of allergens. Green areas represent a positive association between almond allergens and other allergens of the respective sources.

		Possible Cross-Reaction Source								
Source	Allergen	Mahleb	Peanut	Chestnut	Hazelnut	Walnut	Peach	Pollen	Profilin-Containing Plants	Maze
**Almond**	Pru du 3			[65]						
	Pru du 6	[66]								[67]
	Pru du 1							[59]		
	Pru du 4								[68]	
	Pru du γ-conglutin		[69]							

**Table 2:** Almond allergens and their biological function, molecular weight, food processing effects and clinical relevance.

Allergen	Biochemical Name	WHO-IUIS	Isoallergen and Variants	GenBank Nucleotide	UniProt	Biological Function	MW (kDa)	Processing	Clinical Relevance	References
Pru du 3	non-specific Lipid Transfer Protein 1 nsLTP1	Yes (2009)	Pru du 3.0101	FJ652103	C0L0I5	Non-specific lipid transfer protein (nslTP1) and plant defense proteins against pathogens	9	Very resistant to pH, thermal and enzyme treatments	Systemic and life-threatening symptoms; cross reactivity among *Rosaceae* fruit	[112]
Pru du 4	Profilin	Yes (2006)	Pru du 4.0101 Pru du 4.0102	AY081850 AY081852	Q8GSL5 Q8GSL5	Actin-binding protein for cellular function	14	Unstable during heat processing	Mild symptoms and mainly in oral cavity	[68]
Pru du 5	60S acidic ribosomal protein P2	Yes (2007)	Pru du 5.0101	DQ836316	Q8H2B9	Protein synthesis	10	Unknown	Unknown	[86]
Pru du 6	Amandin, 11S globulin legumin-like protein	Yes (2010)	Pru du 6.0101 Pru du 6.0201	GU059260 GU059261	E3SH28 E3SH29	Major storage protein	360	Stable to dry heat but can be denatured by boiling	Severe IgE allergic reactions	[82]
Pru du 8	Antimicrobial seed storage protein	Yes (2018)	Pru du 8.0101	MH922028	A0A516F3L2	Antimicrobial and seed storage function	31	Unknown	Unknown	[90]
Pru du 10	Mandelonitrile lyase 2	Yes (2019)	Pru du 10.0101	AF412329.1	Q945K2	Highly efficient catalytical enzyme	60	Resistant to enzyme digestion	Unknown	[87,89]
Pru du γ-conglutin	Cupin superfamily	No	_______	_______	_______	7S vicilin storage protein	45 for each subunit	Unknown	Unknown	[92]
Pru du 1	PR-10 protein	No	_______	_______	_______	Plant pathogenic and stress response	17	Wet heat processing reduces IgE reactivity	Unknown	[99]
Pru du 2	PR-5/thaumatin-like protein	No	_______	_______	_______	Pathogenic response	23–27	Resistant to protease, pH or heat treatment	Unknown	[113]
Pru 2S albumin	Prolamin super family	No	_______	_______	_______	Seed storage protein	12	Stable to heat treatment	Unknown	[92]

**Table 3:** Example of commercial immunological kits for almond detection and/or quantification and their main characteristics: time for results including extraction times, assay type, limit of detection (LOD), limit of quantification (LOQ) and their manufacturers.

Kit ^1^	Assay Time	Assay Type	LOD (ppm)	LOQ (ppm)	Company
ELISA-based					
MonoTrace ELISA kit	40 min	Monoclonal antibody-based ELISA	0.15	1	BioFront Technologies, Tallahassee, FL, USA
SENSISpec ELISA almond	75 min	Sandwich enzyme immunoassay	0.2	0.4	Eurofins Technologies, Budapest, Hungary
RIDASCREEN FAST Mandel/Almond	50 min	Polyclonal antibody specifically for almond protein detection, sandwich ELISA	0.1	2.5	R-Biopharm AG, Madrid, Spain
AgraQuant^®^ Plus Almond	30 min	Sandwich enzyme-linked immunosorbent assay	0.5	1	Romer Labs^®^, Getzersdorf, Austria
**LFD-based**					
AgraStrip^®^ Almond	11 min	Lateral flow device	2	__________	Romer Labs^®^, Getzersdorf, Austria
Reveal 3-D Almond Test	10 min	Lateral flow device	5	__________	Neogen Corp., Lansing, MI, USA
Lateral Flow Almond incl. Hook Line ^2^	10 min	Lateral flow device	1	__________	R-Biopharm AG, Madrid, Spain

^1^ Mention of commercial kits and trade names is only for exemplification purposes and the authors declare no competing financial interest. ^2^ The hook line is included with the purpose of overcoming the hook effect—very high amounts of an analyte in the sample can lead to falsely lowered or negative results.
